# Glial Cells as Key Mediators in the Pathophysiology of Neurodegenerative Diseases

**DOI:** 10.3390/ijms27020884

**Published:** 2026-01-15

**Authors:** Katarzyna Bogus, Nicoletta Marchesi, Lucrezia Irene Maria Campagnoli, Alessia Pascale, Artur Pałasz

**Affiliations:** 1 Department of Histology, Faculty of Medical Sciences in Katowice, Medical University of Silesia, 40-055 Katowice, Poland; kbogus@sum.edu.pl (K.B.); apalasz@sum.edu.pl (A.P.); 2 Department of Drug Sciences, Pharmacology Section, University of Pavia, Viale Taramelli 14, 27100 Pavia, Italy; nicoletta.marchesi@unipv.it (N.M.); lucreziairenem.campagnoli01@universitadipavia.it (L.I.M.C.)

**Keywords:** glial cells, neurodegeneration, Alzheimer’s disease, Parkinson’s disease, amyotrophic lateral sclerosis, Huntington’s disease, multiple sclerosis

## Abstract

Neurodegenerative disorders are characterized by progressive neuronal loss and dysfunction, yet increasing evidence indicates that glial cells are central mediators of both disease initiation and progression. Astrocytes, microglia, and oligodendrocyte lineage cells modulate neuronal survival by regulating neuroinflammation, metabolic support, synaptic maintenance, and proteostasis. However, dysregulated glial responses, including chronic microglial activation, impaired phagocytosis, altered cytokine production, and mitochondrial dysfunction, contribute to persistent inflammation and structural degeneration observed across Alzheimer’s disease, Parkinson’s disease, amyotrophic lateral sclerosis, Huntington’s disease and multiple sclerosis. Recent advances in single-cell and spatial omics have revealed extensive glial heterogeneity and dynamic shifts between neuroprotective and neurotoxic phenotypes, emphasizing the context-dependent nature of glial activity. This review summarizes current knowledge regarding the multifaceted involvement of glial cells in neurodegenerative disorders.

## 1. Introduction

**Figure 1:**
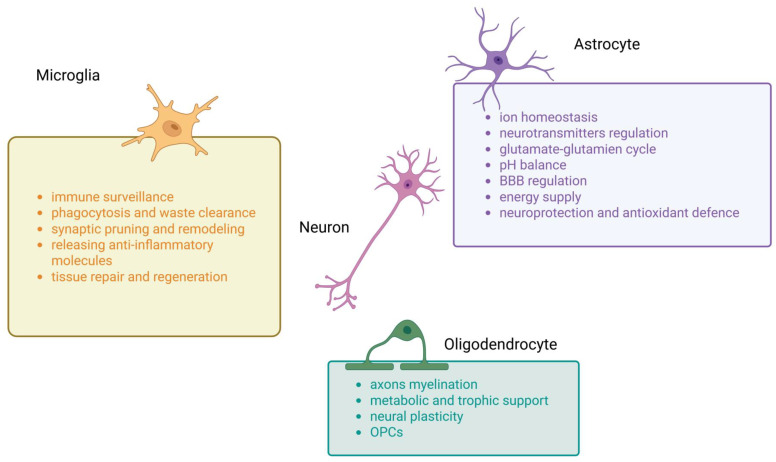
Main functions of microglia, astrocytes and oligodendrocytes in central nervous system (CNS). This summary figure shows the common functions of three types of glial cells. BBB—blood–brain barrier, OPCs—oligodendrocyte progenitor cells.

The era when the neuroglia was considered a relatively inert and physiologically limited structural scaffold of the brain is definitely over. Nowadays, the highly dynamic development of advanced cellular and neuromolecular techniques has allowed us to realize the wide spectrum of mutual interplay between neurons and glia. It is now firmly established that the formation, maturation and functioning of all neurons is fundamentally dependent on these multifaceted and constant interactions (Figure 1). Glial cells exist in several morphological forms known since the 19th century: astrocytes, oligodendrocytes, ependymal cells (or ependymocytes), and microglia. The structural characterization of neuroglia stems from the pioneering work of outstanding neuroscientists, Santiago Ramon y Cajal and Camillo Golgi, and their invention of the technique for impregnating the nervous tissue with silver and gold salts. The morphological description of glial cells they presented remains relevant to this day. Most neuroglia is of ectodermal origin, with the exception of microglia, which is derived from the mesoderm–specifically, the fetal macrophages that migrate into the neural tube during embryonic development [1].

Notably, a special type of astrocyte, called the radial glial cells, appears to be crucially important in both brain organogenesis and adult neurogenesis. In this review article, we will focus on the characteristics of these aforementioned glial cell types in the context of their involvement in the pathogenesis and course of common neurodegenerative diseases, which constitute a serious problem in contemporary neurology.

## 2. Glial Cells

### 2.1. Astrocytes

Astroglia (also known as astrocytes) are a class of neural cells of ectodermal, neuroepithelial origin that maintain homeostasis and provide protection to the central nervous system (CNS) [1]. Astrocytes exhibit a rather heterogeneous morphology across different brain structures [2,3], a variability determined by the cytoarchitecture of the particular cortical or subcortical region. Furthermore, the astrocyte phenotype changes dynamically in both physiological and pathological conditions [4,5,6]. Despite this heterogeneity, the main physiological features of astroglial cells are similar to some extent, as they are specialized to maintain local homeostasis for the brain neural populations. In the CNS, astrocytes are integrated into cellular networks, forming particular areas of adjacent membranes with neighboring cells. These areas contain hundreds of intercellular channels that enable the transport of ions, secondary messengers, and other biologically active molecules [1]. Moreover, the microcirculation in the brain neuropils is also an important part of this ordered network, which includes thousands of synapses. This unique and highly ordered arrangement of astrocytes is often known as an astrocyte microdomain.

Astrocytes are the ubiquitous glial cells of the CNS. They form extensive connections with neurons and probably contact all other brain cell types, possessing a wide range of competencies, supporting the functions of other glial cells in many processes. The most important functions performed by astroglia, including ion homeostasis, neurotransmitter removal, lipid neurochemistry, synapse formation/pruning, synaptic transmission and participation in neurovascular coupling [2] will be outlined below (Figure 1).

Astrocytes not only regulate cerebral circulation in response to neuronal activity, but also play an active role in the production, delivery, utilization, and storage of energy in the brain [7]. Moreover, they express a large pool of membrane receptors and release various gliotransmitters, playing an important role in CNS development, neuronal differentiation/neurite growth, integrating and processing synaptic information, and ultimately regulating synaptic transmission and brain plasticity [8,9,10,11]. Astrocytes are an integral part of the synapse; their processes surround and seal the synaptic cleft, forming a complex structure called the ‘tripartite synapse’.

Astrocytes not only regulate embryonic and adult neurogenesis but can also serve as neuronal progenitors themselves, specifically as the radial glia. Numerous studies over the past few years indicate that astrocytic cellular activity is based on changes in the concentration of calcium ions in the cytosol, rather than on electrical changes in the cell membrane, which instead is typical of neurons, and their excitability is precisely regulated by cellular Ca^2+^ levels [12,13,14].

Numerous studies confirm the key function of astrocytes within synapses, such as the prevention of glutamate excess and regulation of neuronal excitability by uptaking synaptic glutamate [15]. This process is regulated by the glutamate transporter excitatory amino acid transporter 2 (EAAT2)/glutamate transporter 1 (GLT-1) (in rodents and humans, respectively). Astrocytes convert glutamate into glutamine, which is released and taken up by neurons for the synthesis of neurotransmitters such as gamma-aminobutyric acid (GABA) or glutamate. Additionally, various factors released by astrocytes have been shown to promote the formation and maturation of both excitatory and inhibitory synapses [16,17].

In addition to participating in the transmission of information between nerve cells, astrocytes also actively respond to neurotransmitters and neuromodulators. For instance, the activation of astrocytic β-adrenergic receptors (β-AR) by norepinephrine (NE) has been shown to dilate astrocyte protrusions and increase their volume in areas of sensory cortex [18,19,20].

It is currently well known that astrocytes play an active role in the supply, production, utilization, and storage of brain energy [21]. There is a close relationship among brain activity, glutamatergic neurotransmission, energy demand, and glucose metabolism [22,23]. Astrocytes possess unique cytoarchitectonic and phenotypic features that make them ideal for analyzing the brain microenvironment and dynamically responding to subtle biochemical changes in neural networks. They carry out two different types of processes: on the one hand, they are an essential component of the ‘tripartite synapse’; on the other hand, their protrusions are in contact with the interstitial microcirculation. Astrocytes are therefore ideally suited for sensing neuronal activity at synapses and responding to the metabolic demands of neurons [7,24]. They also regulate brain water balance via aquaporins [25].

Astrocytes are also able to control several non-neuronal brain cell populations by releasing various chemokines that attract these cells to specific tissue territories. In this manner, they coordinate the movement of microglia during inflammation and oligodendroglial cells during brain development. Numerous regulatory factors released by astrocytes, such as platelet-derived growth factor (PDGF), leukaemia inhibiting factor (LIF), neurotrophin-3 (NT-3), neurotrophin-4 (NT-4), ciliary neurotrophic factor (CNTF) and insulin-like growth factor 1 (IGF-1), promote the differentiation, proliferation, and survival of oligodendrocyte precursor cells, as well as assisting in myelin formation and axonal remyelination after injury [26].

Numerous studies have demonstrated the dualistic morphology and function of astrocytes depending on the physiological state of brain tissue. On the one hand, they secrete anti-inflammatory factors, thereby facilitating tissue repair and regeneration. On the other hand, they can produce pro-inflammatory cytokines, reactive oxygen species and other neurotoxic substances, thereby intensifying the neuroinflammatory response and sustaining the process of neurodegeneration. On this basis, two types of astrocytes have been identified. The first type, which inhibits the long-term regenerative processes of nervous tissue (A1), while the second type has a positive effect on the regeneration of damaged tissue (A2) [12].

### 2.2. Oligodendrocytes

The primary role of oligodendrocytes is to synthesize myelin and ensheath axons with it during the continuous process known as myelination. Oligodendrocytes not only electrically insulate axons but also influence the formation of sodium channel clusters at the nodes of Ranvier, which enable the efficient propagation of the action potential [27]. Myelination is a process that requires a considerable amount of energy, especially during the development of the nervous system. Oligodendrocytes meet this metabolic demand through the oxidation of glucose and fatty acids. Furthermore, in adulthood, myelin sheaths undergo continuous remodeling to maintain a fine balance between myelin synthesis and degradation [28,29].

Axons require a continuous supply of energy substrates to ensure adequate signal conduction. Studies have revealed that oligodendrocytes express the monocarboxylate transporter 1 (MCT 1) lactate transporter [28,30,31]. Thus, the delivery of energy substrates—such as lactate, glucose, and ketones-to axons is another vital function of oligodendrocytes [32,33].

In addition to their close structural and functional interactions with axons, oligodendrocytes also exhibit numerous interactions with other glial cells. For example, they communicate with astrocytes both through direct intercellular contact and via secreted cytokines, chemokines, exosomes, and signaling molecules [34]. An increasing number of studies indicate that cells of the oligodendrocyte lineage are capable of responding to various neurotransmitters (Figure 1). For instance, glutamate released by the axons of glutamatergic neurons may act as a regulator of oligodendrocyte precursor cell (OPC) number [28].

### 2.3. Microglia

Microglia are highly mobile, mesoderm-derived, surveillance-functioning macrophages found in the brain, functioning as the primary surveillance cells and forming a significant component of the glial CNS [35,36,37].

Although microglia are primarily known for their role in the phagocytosis of pathogens and cellular debris during the immune response, and from changes in inflammatory phenotypes detected in neurodegenerative diseases, they are also involved in numerous non-immunological aspects of CNS development, homeostasis, and repair [38] (Figure 1).

In a state of neural tissue equilibrium, microglia are typically seen as mature, ramified (or branched), and dynamic cells that actively monitor their local microenvironment [38]. Conversely, various morphological forms of microglia are associated with neurodegenerative diseases. In such pathological states, it is possible to observe reactive microglia, which are amoeboid cells characterized by an increased expression of pro-inflammatory proteins and antigen-presenting markers. Morphological changes in microglia have also been found in the ageing nervous system, where the cells develop enlarged bodies and thick processes (protrusions) and tend to accumulate cellular debris [38,39].

It is also common to divide microglia into two phenotypes, M1 and M2, which differ significantly in the functions they perform within the nervous system [40]. The phenotype adopted by microglia appears to be dependent on extracellular stimulation [41]. M1 microglia are pro-inflammatory cells, activating under the influence of lipopolysaccharides (LPS) or interferon-γ (INF-γ). This activation results in the robust secretion of pro-inflammatory cytokines and mediators, including tumor necrosis factor α (TNF-α), interleukin 1 beta (IL-1β), and IL-6 [42]. M2 microglia, in contrast, are cells characterized by anti-inflammatory effects and are sensitive to cytokines such as IL-4 and IL-13. Activated M2 cells secrete IL-10, transforming growth factor-beta (TGF-β), and brain-derived neurotrophic factor (BDNF), which suppress inflammatory processes and promote neural cell growth and repair [43].

Microglia are closely associated with neurons, astrocytes, and blood vessels [44]. Indeed, a recent study reports that microglia interact and communicate with vascular endothelial cells, which form the blood–brain barrier (BBB). This interaction is particularly critical for regulating the entry of solutes, chemicals, and foreign antigens into the brain parenchyma [45].

During brain development, microglia play a major role in the developmental pruning of unnecessary synapses [46]. In the adult brain, ‘branching’ microglia are not quiescent; instead, they constantly surveil the neural tissue. They perform an immune surveillance function and can sense subtle changes in the microenvironment through various surface receptors [36,47]. These cells also release neurotrophic growth factors to promote neuronal survival and enhance neurogenesis. However, during brain injury or degeneration, microglia become activated and release neuroinflammatory factors, growth factors, chemokines, prostaglandins and reactive free radicals [48,49,50].

As previously mentioned, activated microglia provide innate immunity to the CNS by releasing a number of immunocompetent molecules and chemokines, such as macrophage inflammatory protein 1α (M1P1α), various interleukins, and TNF-α. These molecules not only control inflammation but also modulate the brain’s immune response. At the same time, activated microglia also promote neuroprotection by releasing anti-inflammatory and growth factors, including nerve growth factor (NGF), BDNF and NT-3. The overall contribution of microglia to neuronal damage or protection is complex and depends on factors such as the nature and duration of the injury and the age of the patient [51,52].

Although the neurotoxic or destructive potential role of microglia is well known, activated microglia also serve as a defense mechanism against various brain injuries. These cells phagocytose invading microorganisms, remove cellular debris and facilitate tissue repair post-injury. As the resident immune cells of the CNS, they protect the tissue, repair damage, and promote the healing process [53,54]. Beyond their conventional neuromodulatory function, they also contribute to neuroendocrine regulation as well as neurogenesis [55,56].

Although microglia and astrocytes have been historically classified into binary phenotypes (e.g., M1/M2, A1/A2), recent single-cell and spatial transcriptomic studies have clearly demonstrated that glial activation states exist along a dynamic, multidimensional continuum. In this review, we use these dichotomies as simplified conceptual models to facilitate comparison across diseases, while acknowledging that in vivo glial phenotypes are highly heterogeneous, plastic, and context-dependent.

## 3. Glial Cells in Neurodegenerative Diseases

### 3.1. Alzheimer’s Disease

Alzheimer’s disease (AD) is a progressive neurodegenerative disorder and the most common cause of dementia [57]. It is characterized by cognitive dysfunction, memory loss, and motor abnormalities. The key pathological hallmarks of AD are the extracellular accumulation of amyloid-β (Aβ) peptides and the formation of intraneuronal neurofibrillary tangles (NFTs), which ultimately lead to neuronal death and synaptic loss [58]. AD is conventionally categorized into two main forms: the rare familial AD (FAD, early-onset) and the highly prevalent sporadic AD (SAD, late-onset). Aging represents the primary risk factor for AD, with prevalence increasing dramatically with advanced age [59]. While the exact etiology remains unknown, both genetic and environmental factors are believed to play a role [60]. The sporadic form is associated with various comorbidities, including diabetes, hypertension, and poor diet [61]. Recent research suggests that, beyond Aβ and NFTs, neuroinflammation constitutes a third core pathology, playing a significant role in the disease’s development. This highlights the urgent need for effective preclinical diagnosis and therapeutic strategies to prevent or slow the disease before the onset of clinical symptoms [58,62].

Neuroinflammation is a key factor in the initiation, pathophysiology, and progression of AD [63]. This term refers to the inflammatory response occurring within the CNS, primarily characterized by the accumulation and activation of glial cells, mainly astrocytes and microglia, in response to pathology or injury [64]. In the early stages of AD, these brain immune cells provide a degree of neuroprotection [65]. However, as the disease advances, glial cells become chronically activated, leading to the elevated production of pro-inflammatory cytokines linked to oxidative stress. This sustained neuroinflammatory response can cause neurotoxicity and exacerbate Aβ and tau pathologies through multiple mechanisms [64].

Neuroinflammation in AD is triggered by a variety of intrinsic and extrinsic factors. Internally, an individual’s sex, age, and genetics play a crucial role. For example, females face a higher risk due to changes in estrogen receptors, which typically provide a neuroprotective effect by reducing oxidative stress and inflammation [66]. Genetic mutations, such as those in apolipoprotein E4 (ApoE4) and triggering receptor expressed on myeloid cells 2 (TREM2), can negatively impact neuroinflammation and disease trajectory [67,68]. External factors that contribute to neuroinflammation include chronic stress, which exacerbates AD symptoms by activating the hypothalamic–pituitary–adrenal (HPA) axis and increasing cortisol levels. This, in turn, leads to enhanced Aβ and tau aggregation and reduced cognitive abilities [69]. Heavy metal toxicity and oxidative stress are also significant factors, as they can induce protein misfolding and DNA damage, ultimately leading to the activation of glial cells and the release of pro-inflammatory cytokines [70,71]. While a large volume of research exists on neuroinflammation, this review aims to synthesize an overview of the glial cells’ role in AD-associated neuroinflammation, focusing on the chronic activation of microglia and astrocytes as the principal actors.

The age-related decline of glial function results in a state of chronic, low-grade neuroinflammation that sets the stage for neurodegenerative disease. Aged microglia, in particular, produce increased amounts of inflammatory cytokines and reactive oxygen species (ROS) [72]. This oxidative stress creates a self-perpetuating vicious circle. Oxidative stress acts as both a trigger and a consequence of Aβ accumulation and mitochondrial impairment [72]. The continuous generation of ROS further damages glial mitochondria, thereby hindering their ability to provide metabolic support and clear protein aggregates [73]. This results in a dysregulated microglial environment that is prone to cytotoxic activation and less efficient at clearing Aβ, consequently leading to its accumulation [72]. In essence, aging weakens the brain’s immune and homeostatic support systems, allowing the primary pathologies of neurodegenerative diseases to gain a foothold and spread unchecked, ultimately resulting in large-scale neuronal death.

In the context of neurodegenerative diseases like AD, microglia become activated. Initially, this activation can be protective, as microglia attempt to clear harmful substances like Aβ plaques through phagocytosis. They also secrete neuroprotective factors. However, prolonged exposure to Aβ and other pathological signals eventually leads to chronic activation and microglial dysfunction. This pathological state becomes detrimental: microglia begin releasing high levels of pro-inflammatory cytokines (such as IL-6 and TNF-α), chemokines, and ROS, which can directly harm neurons and synapses. This sustained inflammation impairs the microglia’s ability to clear Aβ, leading to a vicious cycle of plaque accumulation and worsening neuroinflammation. The morphology of microglia also shifts, with their branches shortening, making them less efficient at their homeostatic functions.

Various models and experimental conditions have established the purinergic P2X7 receptor (P2X7R) as a pivotal target in microglia activation. For instance, the stereotaxic injection of Aβ into the hippocampus of P2X7R knock-out mice failed to activate microglial cells [74]. Furthermore, the selective P2X7R antagonist GSK 1482160A was successfully used in a hAPP transgenic mouse model (J20) to increase microglial migration and phagocytic capacity, thereby reducing neuroinflammation [75]. Consequently, P2X7R inhibition is proposed as a promising therapeutic approach to reduce neuroinflammation in AD.

Microglia activation is mediated by several mechanisms, not only via P2X7R, but also the receptor for advanced glycation end-products (RAGE) plays a significant role in microglia activation and neuroinflammation in AD [76]. With regard to Aβ transport, RAGE is upregulated in AD and facilitates the transport of Aβ from the bloodstream into the brain [77]. In microglia, RAGE upregulation induces mitochondrial damage, oxidative stress, and the subsequent release of pro-inflammatory cytokines such as IL-1β and TNF-α [78]. Furthermore, the interaction between RAGE and its ligand, High Mobility Group Box 1 (HMGB1), further activates inflammatory pathways like nuclear factor kappa-light-chain-enhancer of activated B cells (NF-κB) [79,80].

Several foundational and recent studies have established the critical role of the microglial NOD-like receptor pyrin domain-containing protein 3 (NLRP3) inflammasome in AD pathogenesis. The crucial work performed by Heneka and coworkers demonstrated that knocking out the NLRP3 or caspase-1 genes (*Nlrp3*−/− or *Casp-1*−/−) in a mouse model of AD (APP/PS1 mice) resulted in reduced Aβ plaque deposition, decreased IL-1β levels, and ameliorated cognitive deficits compared to AD mice with intact genes. This strongly suggests that NLRP3/caspase-1-mediated inflammation is essential for AD progression [81].

In AD, astrocytes become activated and undergo significant conformational, transcriptional, and functional changes, a process collectively known as astrogliosis [82]. These reactive astrocytes exhibit morphological hypertrophy and typically overexpress glial markers such as glial fibrillary acidic protein (GFAP). Mirroring the response of activated microglia, reactive astrocytes increase their production of a wide spectrum of inflammatory mediators, including TNF-α and IL-1 [83]. This inflammatory output initiates a harmful cascade that culminates in the impairment of neuronal functions and accelerated neurodegeneration [84].

Crucially, the reactive astrocyte state is highly heterogeneous and can be broadly classified into detrimental (A1) and beneficial (A2) phenotypes, based on the initiating signals and subsequent functional changes. Furthermore, research has identified a specific cocktail of inflammatory signals released by activated microglia—IL-1α, TNF-α, and complement component 1q (C1q)- that serves as a definitive trigger for inducing the highly detrimental, neurotoxic A1 astrocyte phenotype. This phenotypic conversion, driven by microglial crosstalk, significantly amplifies chronic neuroinflammation in the AD brain [84].

The neurotoxic A1 phenotype actively acquires harmful features, losing crucial neurotrophic and homeostatic functions while gaining neurotoxic properties. This shift is a major contributor to synaptic dysfunction and neuronal death in AD. In contrast, A2 phenotype astrocytes carry beneficial or repair functions by significantly upregulating the secretion of numerous neurotrophic factors that promote neuron survival, growth, and synaptic repair [85].

Reactive astrocytes also regulate microglial function, aiming to promote beneficial outcomes in terms of clearance, inflammation and intercellular connections. Regarding clearance, astrocyte-specific IL-3 acts on microglial receptors, significantly enhancing microglial motility and their capacity to clear plaques [86]. Astrocytes utilize physical structures called nanotubes to establish direct contact with microglia, facilitating the intercellular transfer of aggregated pathological proteins for efficient breakdown and removal [87].

A key feature of AD is the dramatic reduction in the expression and function of the astrocytic glutamate transporter EAAT2. This failure results in pathologically high levels of extracellular glutamate, as demonstrated by Simpson and coworkers in a cohort of AD patients and brain aging, causing chronic neuronal overstimulation and widespread neuronal death, known as excitotoxicity [88]. This chronic exposure severely impairs the astrocyte’s primary antioxidant defence: glutathione (GSH) production. Reduced GSH levels in astrocytes can enhance neurodegeneration as these cells become inflammatory cells and neglect their neuroprotective function [89]. The resulting increase in ROS activates the RAGE-NF-κB pathway in astrocytes. This cascade sustains the pro-inflammatory response by promoting cytokine production and amplifying detrimental interactions with microglia [90].

The neuroinflammation-mediated disruption of the BBB by activated astrocytes is a cascade of events that ultimately compromises the CNS homeostasis and contributes to disease progression. Activated/reactive astrocytes, which are a critical component of the neurovascular unit (NVU), contribute to BBB breakdown primarily through the secretion of inflammatory mediators and structural changes to the NVU. Regarding the secretion of inflammatory and vasoactive mediators, the reactive astrocytes release a variety of factors that directly affect the endothelial cells (ECs) of the BBB. Key examples include pro-inflammatory cytokines and chemokines such as IL-1β, TNF-α, and IL-6 that can signal to the ECs, leading to the disassembly of tight junction (TJ) proteins (e.g., claudins, occludin) that normally seal the gaps between ECs. This action increases paracellular permeability [91]. Moreover, astrocytes can release matrix metalloproteinases (MMPs), which are enzymes that degrade the extracellular matrix (basement membrane) and the TJ proteins, thereby further weakening the structural integrity of the BBB. Vascular endothelial growth factor A (VEGF-A) also functions as a pro-inflammatory factor because it is a potent endothelial permeability agent that can increase the leakiness of the cerebral vessels.

### 3.2. Parkinson’s Disease

Parkinson’s disease (PD) affects more than 10 million people worldwide and ranks as the second most common neurodegenerative disorder [92]. Clinically, PD is characterized by typical motor symptoms such as bradykinesia, rigidity, resting tremor and postural instability. Non-motor symptoms include sleep disturbances, cognitive impairment, autonomic dysfunction and neuropsychiatric issues [92,93,94]. These collective symptoms significantly impact not only the patient’s quality of life but also that of their caregivers. A direct histopathological marker of PD is the highly selective degeneration of dopaminergic neurons located in the *substantia nigra* of the midbrain. This process subsequently causes a decrease in dopamine levels within the striatum [95]. Additionally, the presence of Lewy bodies—intracellular aggregates of abnormally folded α-synuclein—is a characteristic pathological feature of the disease [96]. The exact etiology of PD remains unclear, but multiple factors such as pesticide exposure, genetic predisposition, oxidative stress, mitochondrial dysfunction and ageing are believed to contribute to its onset [97].

Genetic factors are considered major contributors to PD. Mutations or polymorphisms within the α-synuclein (*SNCA*) gene lead to the misfolding and excessive deposition of α-synuclein proteins and the subsequent formation of Lewy bodies [98]. In turn, mutations within the *Parkin*, *PINK1* and *PARK7* (*DJ-1*) genes cause mitochondrial dysfunction by impairing critical quality control pathways like mitophagy, resulting in high levels of oxidative stress and leading to apoptosis [99]. This cascade ultimately involves damage and death of dopaminergic neurons [100]. As studies have demonstrated, mutations in different genes can cause PD characterized by distinct phenotypes. For example, mutations in the *LRRK2* (*Leucine-Rich Repeat Kinase 2*) gene are among the most common in familial PD. This specific mutation promotes mitochondrial dysfunction and is closely associated with neuroinflammation. Research using the *LRRK2* mutation model has shown a significant reduction in the expression of angiotensin-converting enzyme 2 (ACE2). Indeed, ACE2 expression is intrinsically linked to neuronal degeneration and the exacerbation of inflammation within the *substantia nigra*, as this enzyme is generally involved in regulating the renin-angiotensin system that modulates neuroinflammation [101]. These findings underscore the multifactorial etiology of PD and point to the need for more personalized therapeutic strategies [102]. As previously mentioned, one of the main pathological markers of PD is the abnormal folding and deposition of α-synuclein proteins. Under physiological conditions, these proteins occur in neurons as soluble monomers [103], but in PD, they aggregate to form deposits known as Lewy bodies. As recent studies indicate, this accumulation of α-synuclein can activate toll-like receptor 4 (TLR4) receptors on microglia [104,105]. The activation of both microglial and neuronal TLR receptors initiates a cascade of pro-inflammatory responses. This leads to the release of cytokines and nitric oxide (NO), which exacerbates cellular stress [95,106]. Furthermore, this cascade can reduce caspase-8 activity, resulting in necroptosis rather than traditional apoptotic processes [107]. Activated microglia sustain cerebral inflammation by shifting towards a pro-inflammatory M1 phenotype, thus determining an augmented secretion of IL-1β, IL-6 and TNF-α [106]. The α-synuclein aggregates not only have a disruptive effect on neurons but also significantly affect neighboring astrocytes. These cells, responding both to microglial cytokines and to α-synuclein deposits, undergo astrogliosis, transitioning into a reactive state. Indeed, reactive astrocytes secrete cytokines and chemokines, thereby contributing, along with microglia, to the maintenance of a neuroinflammatory state that is destructive to nerve cells [108]. As mentioned, reactive astrocytes are classified into two subtypes A1 and A2 [109]. Subtype A1 astrocytes are characterized as glial cells that act deleteriously on other brain cells, while subtype A2 is reported to act protectively on neurons [110]. Moreover, A1 astrocytes lose their capacity to support neuronal function and participate in synaptic transmission, while simultaneously gaining the ability to induce both neuronal and oligodendrocyte death [109].

It was recently pointed out that the accumulation of α-synuclein in oligodendrocytes occurs in PD, which not only impairs myelin production but also disrupts axonal energy metabolism [111]. Furthermore, incorrect functioning and stunted differentiation of OPCs are considered both potential causes and effects of PD [112].

Currently, PD remains an incurable disease. The available drug therapies (e.g., levodopa and dopamine agonists) primarily alleviate motor symptoms but do not alter the disease’s clinical course [113]. The search for effective therapies has thus intensified the focus on understanding the mechanisms operating within both neuronal and glial cells that may influence PD pathogenesis [114,115].

Microglia become activated in response to neurodegeneration of dopaminergic cells. This activation is followed by the secretion of pro-inflammatory cytokines, including IL-1β, TNF-α, INF-γ, and IL-6. In turn, prolonged microglial activation contributes to a state of sustained neuroinflammation, which exacerbates neuronal damage and accelerates disease progression [115,116,117]. *Post-mortem* analyses and neuroimaging techniques have revealed elevated expression of TSPO (Translocator Protein, an 18 kDa outer mitochondrial membrane protein) in the *substantia nigra* and striatum of PD patients. This elevated expression is indicative of microglia activation, which increases with disease progression. These findings confirm the involvement of microglia not only in the clearance of dead neurons, but also as cells that actively trigger the degeneration of subsequent dopaminergic neurons [118]. Furthermore, persistent inflammation can disrupt the BBB, allowing immune-competent cells to infiltrate the brain, which further exacerbates inflammatory responses [119]. A significant factor in this process is NF-κB, released by reactive astrocytes, which has a destructive effect on the tight junctions of vascular endothelial cells [120,121]. Excessive amounts of deposited α-synuclein, which activates microglia, appear to be the main cause of this overwhelming cerebral inflammation in PD [97,122]. Astrocytes are also thought to contribute to the maintenance of neuroinflammation in PD through several mechanisms, including the dysregulation of BBB permeability, disorder glutamate metabolism and reduced calcium homeostasis, all of which ultimately affect neuronal survival [123]. Specifically, astrocytes in PD exhibit impaired glutamate uptake, which has been attributed to the observed reduced expression of the EAAT2 transporter. This deficit results in an excess of glutamate in brain tissue, leading to excitotoxicity and exacerbating neuronal degeneration [124]. In addition, studies demonstrate that astrocytes possess the ability to capture and clear α-synuclein aggregates from the extracellular matrix (ECM), thereby contributing to neuronal protection. However, prolonged exposure to α-synuclein, coupled with oxidative stress and chronic inflammation, can ultimately lead to astrocyte dysfunction [123,125]. As studies show, glial cells may undergo metabolic changes in response to neurotoxins, which compromise their ability to provide a supportive environment for neurons [117,126] and may even further exacerbate inflammation and oxidative stress [127].

As the above studies report, microglia and astrocytes are not merely reactive cells responding to CNS lesions, but rather integral players in the development of PD. Their involvement in pro-inflammatory signaling and impaired protein clearance makes them highly promising targets for disease-modifying therapies.

While a key role for oligodendrocytes in the pathophysiology of PD has not been fully demonstrated to date, oligodendrocyte dysfunction is clearly observed, particularly with the reduced expression of myelin genes (MBP, MOG, PLP1) in the early stages of the disease. This dysfunction is hypothesized to contribute to white matter degeneration and synaptic instability, resulting in multiple motor and non-motor symptoms experienced by patients [128].

Knowledge of the metabolic interplay between neurons and glial cells, where glial cells actively participate in meeting the metabolic needs of neurons rather than just providing structural support, may be key to designing more personalized therapeutic strategies. For instance, the metabolic reprogramming of neuroglia may represent a novel target for potential pharmaceuticals.

Ultimately, restoring the normal metabolic functions of glial cells may enhance their neuroprotective abilities and significantly improve PD treatment outcomes [95].

### 3.3. Multiple Sclerosis

Multiple Sclerosis (MS), a disorder of the CNS characterized by inflammation, demyelination, and neuronal loss, is the most common cause of non-traumatic neurological disability in young adults [129]. A critical, early step in MS involves the BBB. This barrier, which normally protects the CNS, becomes compromised due to pro-inflammatory activity. This breakdown makes the BBB permeable, allowing various immune cells (such as T and B cells) to infiltrate the CNS. Once inside the brain and spinal cord, these immune cells release inflammatory mediators, leading to the characteristic lesions and further disease progression [130,131,132]. The early progression of MS is strongly influenced by glial cell activation [133], which results in focal demyelinating white matter lesions with distinct cellular profiles. Active lesions are characterized by the presence of myelin-phagocytosing microglia/macrophages, loss of oligodendrocytes, and the accumulation of pro-inflammatory astrocytes; notably, scar formation is limited [134,135]. Mixed active/inactive lesions (chronic) are characterized by a hypocellular core but retain intense inflammation at the border, which is driven by major histocompatibility complex (MHC)-presenting microglia/macrophages. Although an astroglial scar is present at the core, these lesions demonstrate a crucial capacity to initiate remyelination at their edges [136,137]. In contrast, both inactive lesions and remyelinated “shadow plaques” show a significant reduction or near absence of inflammatory cells. The shadow plaques, in particular, signal successful repair due to the reestablishment of oligodendrocytes and minimal associated axonal damage [138,139].

The pathophysiology of MS involves various cellular components, including astrocytes, microglia, and oligodendrocytes. While the immune roles of astrocytes and microglia are well-established, growing evidence now implicates oligodendrocytes in modulating immune reactivity [140].

Activated astrocytes initiate the release of cytokines and chemokines, which contribute to the development and progression of MS by promoting the rapid death of neurons and oligodendrocytes [110]. Depending on their surrounding microenvironment, astrocytes possess the capacity to secrete either anti- or pro-inflammatory molecules, thereby influencing the dynamic processes of demyelination and remyelination. When activated, astrocytes can release cytokines (TNFα and IL-1β), chemokines (CC Motif Chemokine Ligand 2-CCL2 and interferon gamma-induced protein 10-CXCL10), BDNF, and VEGF, all of which contribute to increased BBB permeability [141,142]. Evidence shows that in MS patients, the perivascular astrocytic end-feet are damaged in early lesions, suggesting that astrocyte injury may be a driver of demyelination [143,144].

Furthermore, astrocytic activation has been shown to promote T cell infiltration into the CNS by expressing vascular cell adhesion protein 1 (VCAM-1) [145], increase the expression of CCL2, contributing to of immune cells recruitment during chronic experimental autoimmune encephalomyelitis (EAE; the primary animal model for MS) [146], cause the upregulation of aquaporin-4 (AQP4) in *post mortem* MS tissue and EAE mouse model [147,148]. During EAE, Th1 and Th17 T cells (key effector cells in CNS autoimmunity) induce the production of granulocyte-macrophage colony-stimulating factor (GM-CSF). This mediator is implicated in microglia activation, ZO-1 transcription downregulation, and expression of adhesion molecules like intercellular adhesion molecule 1 (ICAM-1) and VCAM-1, with consequent neuroinflammation, BBB disruption, and tight junction disassembly [149,150]. Activated astrocytes often impair remyelination through the secretion of inhibitory molecules, extracellular matrix components, and the formation of the glial scar [151]. This inhibition is mediated by several factors, including: endothelin-1, which activates the Notch pathway and disrupts astrocyte-oligodendrocyte communication [152,153]; the release of PDGF and FGF2 [154,155]; the increase in connexin 43, whose depletion accelerates remyelination [156]. However, recent evidence suggests the multifaceted role of astrocytes in remyelination following injury. They act as central organizers of the repair process through various mechanisms, including: removing myelin debris, recruiting OPCs to the lesion (by expressing chemoattractant molecules, such as CXCL1, CXCL8, and CXCL10), promoting OPC proliferation (by secreting PDGF, fibroblast growth factor 2 (FGF2), IGF-1) and supporting the survival of new oligodendrocytes (by upregulating cholesterol synthesis via the Nrf2 pathway) [157,158,159]. Chronic activation of microglia during neurodegeneration leads to the prolonged release of pro-inflammatory molecules, resulting in both detrimental and protective effects depending on the microenvironment [133]. In the context of MS, there is emerging evidence for the direct or indirect implication of microglia in its pathology. Sustained microglial activation promotes both neuroinflammation and neurodegeneration. Specifically, the formation of microglial nodules is associated with more severe MS pathology [160]. Evidence suggests a central role of microglia in driving this damage, as their removal prevents demyelination, oligodendroglial loss, and reactive astrocytosis in models of cuprizone (CPZ)-induced demyelination [161]. Microglia are thus key players in MS progression, acting as agents of both defence and damage. On one hand, they perform the beneficial function of clearing myelin debris via phagocytosis; on the other hand, they release a wide array of pro-inflammatory cytokines (including IL-1β, TNFα, IL-6, and IL-12) and chemokines (including CCL2, CCL4, CCL5, and CCL12) that recruit immune cells and facilitate tissue breakdown [162]. Of note, a growing body of evidence indicates that microglia and T cells colocalize in MS lesions, and their presence correlates with axonal injury [163]. In detail, microglia initiate this damaging cycle by secreting IL-12 and IL-13 to promote T helper 1 (Th1) cells accumulation [164]. Then, Th1 cells drive microglia toward the pro-inflammatory M1 phenotype, thereby escalating the immune response, increasing T cell reactivation, and enhancing infiltration [165,166]. This inflammatory cascade is further amplified by T cell-activated astrocytes, which release factors that enhance microglial movement and activation [167].

Several key molecular mechanisms involving microglia contribute to MS pathology, primarily through pro-inflammatory signalling pathways and regulatory factors. For instance, the colony-stimulating factor 1 receptor (CSF1R), which is essential for microglial survival, and TNFα, a major pro-inflammatory cytokine secreted by activated microglia, are both upregulated in MS models and patient lesions [168,169,170]. Specifically, activated microglia use CSF1R signalling to drive demyelination, especially in progressive MS [161,171]; conversely, CSF1R inhibition has been shown to reduce neuroinflammation and microglial activation in the EAE model [169]. Moreover, TNFα is pivotal in driving microglia toward the damaging M1 phenotype, activating the NF-κB pathway, and enhancing the subsequent release of pro-inflammatory molecules [172,173]. Notably, the inhibition of inhibitory kappa B kinase beta (a key regulatory kinase in the activation of NF-κB) significantly ameliorated disease progression in EAE models by reducing microglial infiltration, M1 polarization, and T cell response [174].

Although microglia contribute to MS pathology, their function is pivotal in orchestrating remyelination. Microglia and macrophages concentrate at the lesion border where remyelination initiates, and their phenotype determines the outcome. High densities of the anti-inflammatory (M2) phenotype are consistently found in areas of active remyelination but are absent in areas where repair fails, underscoring the necessity of this M1-to-M2 transition [175]. Supporting this, M2 polarization has been shown to regulate spontaneous remyelination in a CPZ-demyelination model [176]. Microglia promote remyelination by efficiently clearing myelin debris and secreting regenerative factors; however, persistent activation remains detrimental [177]. These cells facilitate repair by producing desmosterol (a cholesterol precursor), which resolves inflammation and aids myelin synthesis [178]. A key molecular mediator of debris clearance is CX3CR1, which is necessary for internalization [179]. Furthermore, CSF1R knockdown has been shown to lead to impaired myelin clearance and debris accumulation following CPZ-induced demyelination [180]. In addition to debris clearance, microglia orchestrate remyelination by directly modulating OPC behaviour [181]. Their initial pro-inflammatory phenotype recruits OPCs (e.g., via Semaphorin-3F) [177,182], after which a phenotype shift toward an anti-inflammatory state is required to facilitate OPC differentiation and myelin formation [177]. Microglia regulate OPC development by secreting inflammatory factors such as TNFα, IGF1, and IL-1β [183,184,185]. Finally, the observation that microglia depletion impairs OPC differentiation underscores their unique and essential role in successful remyelination [186].

In the context of MS, it has been observed that BBB breakdown is associated with the interaction between blood vessels and oligodendroglia, which in turn triggers CNS inflammation through the activation of microglia and macrophages [187]. Moreover, adhesion molecules such as ICAM-1 and VCAM-1 are upregulated in the EAE animal model; specifically, blocking ICAM-1 can reduce the binding of Th1 cells to oligodendrocytes, suggesting that these molecules mediate critical interactions with CD4+ T cells [188]. The fate of OPCs is dictated by the local inflammatory milieu. The presence of pro-inflammatory mediators and cells, such as effector T cells, IFN-γ, and ROS, actively inhibits the differentiation of OPCs into mature myelin-producing cells, thereby preventing successful remyelination [189]. Conversely, a supportive environment providing appropriate signals for OPC growth and maturation is essential. For instance, fibroblast growth factor (FGF) signalling is vital for regenerating oligodendrocytes and de novo myelin formation. This is supported by the upregulation of FGF2 and its receptor (FGFR) in MS patients and animal models; notably, the loss of the FGF1/FGFR2 signalling pathway significantly impairs myelin recovery following chronic demyelination [190].

It is notable that the pro-inflammatory state of OPCs can simultaneously drive tissue damage and prevent repair. This suggests that inhibiting these inflammatory pathways could mitigate tissue destruction while enhancing the regenerative capacity of OPCs [191]. Successful remyelination hinges on the timely recruitment of OPCs to the lesion site, where they must proliferate and differentiate [192]. While pre-existing mature oligodendrocytes offer limited repair potential, adult OPCs are the primary effectors of axonal re-sheathing. This reparative response is primarily seen in active MS lesions, which typically feature a high concentration of OPCs. However, the most critical factor for OPC differentiation and effective myelin repair is the resolution of local inflammation [193,194]. The remyelination process is finely tuned by a complex balance of glia-derived molecular signals. Astrocytes and microglia initially release a cocktail of chemoattractants (including IGF1, TNFα, PDGF-AA) that successfully recruit and prime OPCs for maturation at the damage site [195]. However, OPC maturation is negatively regulated by other cytokines (IL-10, IL-6, and IFN-γ) [196]. While specific pathways, such as PDGF-A-induced ERK and CXCL12-stimulated MEK/ERK and PI3K/AKT, drive OPC motility and differentiation [197,198], glial cells also release inhibitory factors (chemorepellents and enzymes) that impair OPC recruitment and sustain inflammation [199]. Specifically, IFN-γ acts as a potent inhibitor of remyelination by inducing OPC senescence via the upregulation of the PRRX1 transcription factor, thereby halting their proliferative and reparative capacity [200].

Finally, T cells exert a dual influence on remyelination: while the inflammatory myelin-reactive T cells drive lesion formation and directly inhibit OPCs and repair [191,201]; activated T cells can also be beneficial by secreting growth factors that promote OPC proliferation [202,203].

### 3.4. Huntington’s Disease

Huntington’s disease (HD) is an autosomal dominant neurodegenerative disorder classically defined by the progressive loss of medium spiny neurons (MSNs) within the striatum and cortex, resulting from an expanded polyglutamine (polyQ) tract in the huntingtin protein (mutant huntingtin mHTT) [204]. Although mHTT is ubiquitously expressed in nearly all cell types throughout the central nervous system (CNS), the traditional view long emphasized the intrinsic vulnerability of neurons. However, emerging, rigorous research has fundamentally shifted this paradigm, establishing glial cells—astrocytes, microglia, and oligodendrocytes—not as passive responders to neuronal distress, but as early, active, and causal contributors to the pathogenic cascade [205].

The recognition of glial cells’ critical role stems from evidence demonstrating that these supportive cells both lose essential normal functions (loss-of-function) and gain detrimental neuropathic phenotypes (gain-of-function) in HD.

Astrocytic alterations are recognized as an early and causal component of the disease. In HD, astrocytes transition from their quiescent, supportive state to a reactive morphology, a hallmark of neuroinflammation in the HD brain. Regarding reactive phenotype, astrocytes expressing mHTT develop a progressive phenotype of reactive astrocytes. Studies show that the selective expression of mHTT in astrocytes in mouse models is sufficient to induce changes in their morphology [206]. In addition, astrocytes play an important role in neurotransmitter uptake and recycling, where glutamate is the main excitatory neurotransmitter in the brain. Excitatory amino acid transporters 1 and 2 (GLAST and GLT-1), localized to astrocyte processes can manage around 80% of total glutamate [207], where a decreased expression of GLT-1 is a common feature observed in neurodegenerative disorders [208]. Histological analysis in *post-mortem* brains of HD patients has confirmed the presence of astrogliosis, particularly in the caudate nucleus (the earliest stage). However, some studies suggest that strong evidence of reactive astrogliosis or cell death may not be present at the *earliest* symptomatic ages when other functional losses, such as GLT-1 loss, are already occurring. This implies that functional failure can precede severe morphological changes [209,210,211]. The aforementioned theory has been supported by study showing that wild-type mice treated with a specific pharmacological inhibitor of GLT-1 displayed a similar change in the magnitude of Ca^2+^ signalling. These results also implicate glutamate uptake as a key regulator of astrocyte evolvement to the cortico-striatal circuit activity [210]. Interestingly, restoring Kir4.1 (inwardly rectifying K + channel expressed exclusively in the brain glial cells) levels using a viral-mediated approach also rescues GLT-1 expression in astrocytes, perhaps explaining why Kir4.1-treated R6/2 mice also have partial recovery of their response to evoked cortico-striatal signalling [210]. While changes in Kir4.1 and GLT-1 levels in HD astrocytes clearly play a part in the development of electrical abnormalities in these cells, spontaneous calcium signals are only partially rescued through Kir4.1 and GLT-1 elevation, suggesting that there are likely other molecular changes that contribute to these deficits [212]. Given the exciting finding that restoring some of the homeostatic electrical properties of astrocytes can not only rescue electrical deficits in neurons but also abrogate HD-related behaviours, this will be an important and promising area for future studies.

Not only the downfall of astrocyte homeostatic function but also a detrimental interaction with neuronal circuits is involved in HD pathophysiology. The functional collapse of astrocytes leads to non-cell-autonomous toxicity, meaning the astrocytes fail to protect neurons and actively contribute to their decline. In a direct neuronal impairment, selective expression of mHTT solely in astrocytes in mouse models is sufficient to induce neuronal dysfunction, including a reduction in medium spiny neuron (MSN) formation.

Last but not least, it is important and crucial that the astrocyte-microglia neurotoxic axis, which represents a critical mechanism of non-cell-autonomous neurotoxicity in HD, whereby dysfunctional glia cooperate to actively drive neuronal degeneration [205]. Activated, pro-inflammatory microglia (M1 phenotype) secrete specific factors, including TNF-α, IL-1α, and C1q [213]. This signal cocktail drives the conversion of supportive astrocytes into the neurotoxic A1 reactive astrocyte phenotype. A1 astrocytes secrete neurotoxic factors, establishing a devastating non-cell-autonomous cascade that accelerates neuronal death. This process involves a specific, two-step cascade where the resident immune cells (microglia) initiate a signal that compels the neighboring supportive cells (astrocytes) to turn into a destructive phenotype. Once induced, A1 astrocytes exert dual detrimental effects on surrounding neurons: a severe loss of neuroprotective function and a gain of toxic effects. This establishes a devastating non-cell-autonomous cascade that accelerates neuronal death [110,213]. By initiating this A1 conversion, microglial dysfunction essentially recruits astrocytes into the neurodegenerative process, multiplying the systemic failure of the CNS environment and accelerating the loss of vulnerable neurons [85].

### 3.5. Amyotrophic Lateral Sclerosis

Amyotrophic Lateral Sclerosis (ALS) is a highly debilitating motor neuron disease characterized by the progressive degeneration of both upper and lower motor neurons in the motor cortex, brainstem, and spinal cord. The paralysis typically starts in a focused area, consistent with the initial location of the pathology, and then spreads to adjacent motor neuron pools, suggesting a pattern of local dissemination [214,215]. ALS is traditionally categorized as either sporadic (sALS), which accounts for the majority of cases without a clear family history, or familial. While sporadic implies no family history, it does not mean there is no genetic basis. It is now known that a significant portion of sALS cases have genetic origins: specifically, 1–3% are linked to a mutation in the cytosolic superoxide dismutase 1 (SOD1) gene, 5% or more are caused by intronic expansions in the chromosome 9 open reading frame 72 (C9ORF72) gene, and other, rarer mutations in genes like *FUS*, *TARDBP*, and *OPTN* have also been identified in sALS patients [216,217]. ALS isn’t solely a neuronal disease; it arises from damage that affects motor neurons (MNs) and their surrounding support cells, glial cells. Several studies suggest that glial cells are key drivers in determining when disease symptoms start and how quickly they progress. While much of the early evidence came from mSOD1 research, growing evidence confirms that glial cells contribute to other subtypes of ALS as well [216].

One of the mechanisms involving astrocytes in ALS pathophysiology is dysfunction of EAAT2, which mediates glutamate transport [218]. It has been known that clearing glutamate from excitatory synapses is essential for normal neural transmission, and its impairment leads to damage to neurons [219]. In detail, EAAT2 is cleaved by caspase-3, and these fragments are accumulated within the astrocytic nuclei in the spinal cord [220]. Of note, EAAT2 fragments accumulation coincides with ALS progression [221]. In this way, the reduced number of EAAT2 receptors on membranes causes the impairment of astrocytic ability to buffer glutamate from synapses. Therefore, glutamate accumulates in the synaptic cleft, leading to excessive and pathological neuronal stimulation that disrupts ionic homeostasis. This process, termed glutamate excitotoxicity, contributes to MN damage in ALS [221,222,223].

In neurodegenerative diseases, including ALS, astrocytes undergo significant morphological and functional changes, becoming reactive in response to various stimuli, including soluble factors secreted by microglia [110]. ALS astrocytes display changes in key protein markers: for example, they express selective markers of astrogliosis, but also those typical for immature astrocytes, such as high levels of non-filamentous GFAP [224,225].

In ALS, activated astrocytes become detrimental to MNs, contributing to their damage rather than supporting their survival. This non-cell-autonomous toxicity is consistent across multiple in vitro and in vivo studies using various murine models and human tissue, all demonstrating declined neuronal viability [224,226,227,228,229,230,231]. The damage is mediated by astrocyte-specific soluble factors that are upregulated and secreted by ALS astrocytes. These include several cytokines and growth factors (such as IL-6, CXCL1, CXCL10, CXCL12, TNFα, and TGF-β1) and molecules, like the prostaglandin D2 receptor [230,232,233,234,235,236]. These secreted factors directly alter MN morphology, causing cell bodies to shrink and axons to shorten. More severely, they induce axonal swelling and the formation of mSOD1 and ubiquitin-positive aggregates in the MNs [230,234]. As these aggregates appear even before the onset of ALS symptoms and increase in parallel with reactive astrogliosis, they are tightly linked to disease progression [237]. The cytotoxic mSOD1 is thought to impair mitochondrial function within the MNs, raising intracellular levels of ROS and Ca^2+^, which triggers an inflammatory and nitrosative response [226,227,228,231,238,239]. This mitochondrial damage culminates in the release of pro-cell death factors, driving the MNs toward necroptosis [240]. Similar to the mSOD1 astrocytes, human and murine-derived astrocytes with C9ORF72 or TAR DNA-binding protein 43 (TDP-43) pathology exhibit altered physiological characteristics and negatively impact nearby cells, particularly MNs.

A shared feature between mSOD1 and C9ORF72 astrocytes is the reduced expression of glutamate transporters that causes excitotoxicity in MNs [241]. This has been observed in a C9ORF72 Drosophila model of ALS [242]. Moreover, C9ORF72 astrocytes contribute to MN degeneration through non-cell autonomous effects, specifically by releasing soluble factors and extracellular vesicles containing specific microRNAs. Of note, these microRNAs, upon interacting with targets like semaphorin proteins, induce axonal retraction and worsen overall MN survival [243,244].

The astrocytic factors also influence the function and immune responses of other neighbouring cells, notably microglia [245].

Microglia are consistently activated in all cases of ALS. Several studies confirm that microglial activation occurs specifically at the sites where MN are damaged, both in human ALS patients and in mSOD1 transgenic mouse models [246,247]. Moreover, in the motor cortex, a greater degree of microglial activation is directly linked to the severity of upper MN degeneration signs [246]. In animal models, as ALS progresses, the number of resident microglia increases, and their activation states range along a continuum between two classical functional phenotypes: the neuroprotective (M2) state and the neurotoxic (M1) state [248,249]. Supporting this functional shift, morphological studies have identified distinct microglial types across the disease’s timeline in mSOD1^G93A^ mice [250].

Microglia undergo a critical phenotypic transformation throughout the progression of ALS driven by SOD1 mutations, shifting from a protective state to a toxic one. In the pre-symptomatic stage, microglia exhibit an anti-inflammatory profile. They show an overexpression of IL-10 and have an attenuated response to immune challenges [251]. At the disease onset, microglia change to a neuroprotective M2 phenotype, improving MN survival when grown together in vitro. This is indicated by the upregulation of M2 markers like chitinase-like protein-1 (Ym1) and CD206 in the spinal cords of ALS mice. In the final phase, microglia change to a toxic M1 phenotype that leads to increased neuronal death. These microglia express high levels of NADPH oxidase 2 (NOX2), a prototypic M1 marker [248,252]. Moreover, microglia expressing mutant mSOD1 show increased expression of endoplasmic reticulum stress pathway components, such as CCAAT/enhancer-binding protein (C/EBP0) homologous protein [253], which may contribute to their toxic nature. M1 microglia also appear hyper-reactive to inflammatory stimuli [254], exacerbating the characteristic neuroinflammation of ALS. It has been found that NF-kB, a key regulator of inflammation, is upregulated in the spinal cords of ALS patients and mSOD1^G93A^ mice; however, NF-kB inhibition wasn’t enough to save MNs, while the deletion of NF-kB signalling in microglia successfully rescued MNs from microglial-mediated death in vitro and extended survival in ALS mice by reducing pro-inflammatory activation. Conversely, the constitutive activation of NF-kB in wild-type microglia caused gliosis and MN death both in vitro and in vivo [255]. Another mechanism of damage produced by microglia expressing mSOD1 is the excessive extracellular superoxide production. It has been observed that the mSOD1^G93A^ mutation inhibits GTPase activity, leading to the overproduction of high levels of extracellular superoxide [256,257].

Microglial function is also disrupted in ALS caused by C9ORF72 gene mutations. Evidence suggests that the resulting loss of C9ORF72 protein function contributes to the progression of ALS. For instance, when the C9ORF72 gene is inactivated in mice, the animals display abnormal microglia and age-related neuroinflammation [258,259,260]. This highlights that a non-cell-autonomous, microglia-mediated inflammation likely contributes to ALS pathology.

Microglial activation and MN degeneration are also central features in models involving TDP-43 pathology [261]. Post-mortem studies show that over 90% of ALS patients have cytoplasmic TDP-43 aggregates in the spinal cord [262]. TDP-43 activates microglia by interacting with the CD14 receptor on the cell surface, initiating a pro-inflammatory cascade and resulting in neuronal cytotoxicity. In detail, when activated by TDP-43, microglia express NOX2 and produce pro-inflammatory mediators like TNFα and IL-1 [263]. Despite the negative impacts of microglia, other research points to a positive role for microglia in MN survival [264]. In this regard, Spiller and colleagues proposed that in TDP-43 pathology, it might be more effective to encourage “appropriate” microglia-mediated inflammation to help clear pathological TDP-43 proteins and assist axonal regeneration.

Recent research highlights a growing focus on the roles of oligodendrocyte dysfunction and myelin damage as significant contributors to neurodegeneration across various neurological diseases, notably including ALS [265]. These cells are severely impacted early in ALS pathology, potentially before MN death, as seen in the mSOD1^G93A^ mouse model [266,267]. Although progenitor cells proliferate rapidly, they fail to mature and replace the degenerated oligodendrocytes, leading to demyelination of MN axons [266,267]. Crucially, oligodendrocytes support MNs by providing lactate via the Monocarboxylate transporter 1 (MCT1). However, mSOD1^G93A^ impairs MCT1 expression in oligodendrocytes (a defect also observed in human sporadic ALS), further contributing to MN vulnerability [30]. In fact, reducing mutant mSOD1^G93A^ specifically in oligodendrocytes produces a greater delay in disease onset than that observed in MNs in mSOD1^G37A^ [266,268,269]. Mature oligodendrocytes in the spinal cord of SOD1^G93A^ ALS mice undergo extensive degeneration before any disease symptoms appear [266,267]. Supporting this early, critical role, the selective removal of mutant mSOD1^G93A^ from oligodendrocytes significantly delays disease onset and extends survival [266]. Furthermore, in laboratory settings, oligodendrocytes derived from ALS patients were shown to be capable of inducing MN death when co-cultured, collectively suggesting that the dysfunction of mature oligodendroglia, driven by mSOD1, is a critical factor in ALS pathology [270]. A key study using a transgenic zebrafish model, where mSOD1^G93A^ was confined solely to oligodendrocytes, provided strong evidence that cell dysfunction is sufficient to induce ALS-like MN degeneration. The expression of mSOD1 directly led to oligodendrocyte degeneration, characterized by myelin sheath disruption and loss of the MCT1 transporter, culminating in MN loss. This dysfunction also correlated with behavioural, learning, and motor impairments early in the disease course.

Studies have demonstrated that oligodendrocytes expressing mSOD1^G93A^ can induce electrophysiological changes in healthy MNs, ultimately leading to their death [270]. These changes include increased persistent Na^+^ currents that cause hyperexcitability, as confirmed by Pieri and colleagues [271]. Importantly, this finding, supported by both in vitro and mouse model data, aligns with clinical observations that cortical hyperexcitability is one of the earliest neuronal alterations detected in ALS patients, preceding symptom onset [272,273]. This observation was recently corroborated by work on cultured neurons from neonatal SOD1^G93A^ mice [274]. Oligodendrocytes in *postmortem* tissue from ALS patients were observed to contain TDP-43- and FUS-positive inclusions, indicating a potential dysfunction of autophagy in these glial cells [275]. To determine the clinical relevance of mouse model findings, a comparative in vitro investigation was conducted using both human induced pluripotent stem cell (iPSC)-derived and mouse neural progenitor cell (NPC)-derived oligodendrocytes from ALS- and control-source samples. This study demonstrated that the ability of ALS oligodendrocytes to induce MN toxicity in vitro is not dependent on their cellular origin. While mSOD1 reduction in OPCs successfully abrogated toxicity, the same manipulation in differentiated oligodendrocytes was ineffective. Moreover, the toxicity associated with the C9ORF72 repeat expansion proved to be SOD1-independent and did not involve deficits in lactate release. This evidence strongly suggests that the C9ORF72 mutation delineates a neuropathologically specific subgroup of ALS patients that may require treatment strategies distinct from those targeting mSOD1 pathology [270].

## 4. Conclusions

**Figure 2:**
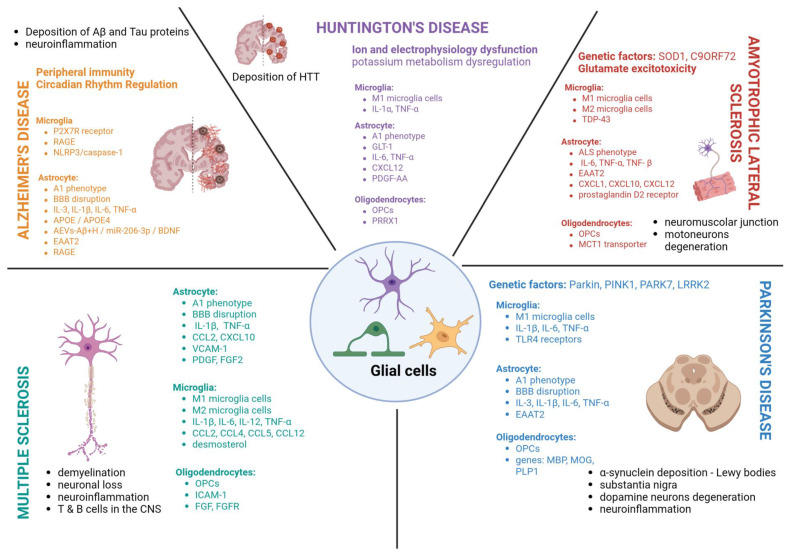
Actions of microglial cells, astrocytes and oligodendrocytes in neurodegenerative diseases—Alzheimer’s disease, Parkinson’s disease, multiple sclerosis, Huntington’s disease and amyotrophic lateral sclerosis.

The complex pathophysiological processes underlying neurodegenerative diseases are associated with abnormal function of both neurons and glial cells, resulting in disrupted interactions between these cell populations. Accumulating studies have revealed a growing body of new data on the physiology of glial cells in the brain during both initial and advanced stages of degenerative changes (Table 1). However, the question remains whether the observed changes in glial cell neurochemistry constitute the primary cause of these conditions or are secondary to the neuronal dysfunction with which neuroglia is inextricably linked. Clarifying this issue appears particularly important for the development of new pharmacotherapeutic strategies based on the selective modulation of the function of specific glial cell populations by targeting their specific receptors and intracellular metabolic pathways. A better understanding of neuroglia–neuron interactions, together with pharmacological progress, may introduce some novel efficient medications, which have only been used in animal model studies so far. Astrocytes seem to be critical players in pathogenesis of all neurodegenerative diseases, especially ALS and MS, across both human and mouse models. Although not all aspects of their function are explained sufficiently, they should be considered a key and promising therapeutic target for future research and drug development. It should also be highlighted that astrocytes can interplay with oligodendrocytes and support myelin formation. Importantly, some possible future remyelinating therapies for all neurodegenerative diseases that target oligodendrocyte precursor cells (OPCs) differentiation or their IFN-γ and mSOD1 signalling must strictly consider all other phenotypes of glial cells. On the other hand, it is recently strongly suggested that several mutations of oligodendrocytes genome, e.g., of C9ORF72 may delineate a neuropathologically specific subgroup of ALS patients that may require treatment strategies distinct from those targeting impaired mSOD1 signalling. The last promising therapeutic strategy is the modulation of the microglia action either by promoting a neuroprotective state or encouraging appropriate microgliosis, e.g., via modulation of microglial purinergic receptors P2X7R to reduce neuroinflammatory processes (Figure 2). To sum up, there are still many questions related to the selective neuroglia pharmacomodulation and many more detailed basic studies are needed to prove its potential usefulness for the efficient and safe treatment of neurodegenerative diseases.

## Figures and Tables

**Table 1:** Summary of the main mechanisms and molecular pathways undertaken by glial cells in Alzheimer’s disease, Parkinson’s disease, Multiple sclerosis, Huntington’s disease and Amyotrophic lateral sclerosis based on multiple tiers. Evidence tier: Tier 1—Human Interventional Evidence, Tier 2—Human Observational & Genetic Evidence, Tier 3—Animal Models, Tier 4—Primary Human or Animal Cell Culture.

Disease	Dominant Glial Mechanisms	Representative Molecular Pathways/Targets	Evidence Tier [References]
**Alzheimer’s disease (AD)**	• Maintaining chronic inflammation • Aberrant synaptic pruning • Astrocyte excitotoxic failure • BBB/neurovascular unit (NVU) disruption	**Microglia:** TREM2–APOE, NLRP3–caspase-1, P2X7R, RAGE–IL-1β/ TNF-α, RAGE-NF-κB **Astrocytes:** EAAT2/GLT-1 loss, A1 astrocyte phenotype (C1q–IL-1α–TNF-α), VEGF-A, MMPs **Neurovascular unit (Activated/reactive astrocytes):** Tight junction loss (claudin-5, occludin)	**Tier 2** [61,62,66,69,91] **Tier 3** [74,75,76,77,81,86] **Tier 4** [74,80,86,90]
**Parkinson’s disease (PD)**	• Microglial-driven neuroinflammation • Astrocytic glutamate metabolism disorder • BBB permeability dysregulation • Calcium homeostasis decreasing • Oligodendrocyte disfunction/OPCs stunted differentiation	**Microglia:** TLR4–NF-κB, NLRP3, LRRK2-ACE2, TSPO **Astrocytes:** EAAT2 downregulation, A1 astrocytes phenotype, cytokine release **Oligodendrocytes:** MBP, PLP1, MOG downregulation	**Tier 1** [113] **Tier 2** [99,101,107,118,128] **Tier 3** [110,116,124,126,127] **Tier 4** [111,112,117,123,127]
**Multiple sclerosis (MS)**	• Autoimmune-driven microglial inflammation • Myelin-phagocytosing microglia • BBB breakdown (early event) • Oligodendrocyte death & remyelination failure • Astrocyte scar formation	**Microglia:** MHC, CSF1R, TNF-α, NF-κB, CX3CR1, IL-12/IL-13-promoting Th1 **Astrocytes:** TNF-α, CCL2, CXCL10, BDNF, VEGF, VCAM-1, ICAM-1, AQP4, endothelin-1–Notch pathway **OPCs:** PDGF-AA, FGF2/FGFR, CXCL12–MEK/ERK, CXL12-PI3K/AKT, IFN-γ–PRRX1	**Tier 2** [134,136,144,147,160,171] **Tier 3** [143,146,148,149,152,153,158,161,169,174,175,176,178,179,180,183,184,185,186,187,190,201,202] **Tier 4** [145,154,155,156,157,159,165,167,173,197,198,200,203]
**Huntington’s disease (HD)**	• Early astrocytic dysfunction • Impaired glutamate and K^+^ homeostasis • Microglial activation and inflammation generation • White-matter/myelin abnormalities	**Microglia:** NF-κB, cytokine release **Astrocytes:** GLT-1/EAAT2 loss, Kir4.1 downregulation, Ca^2+^ signaling defects, A1 phenotype activation **Oligodendrocytes:** myelin gene dysregulation	**Tier 2** [206,209] **Tier 3** [206,210,211,212] **Tier 4** [110,207,208,210,211]
**Amyotrophic lateral sclerosis (ALS)**	• Glial excitotoxicity causes non-cell-autonomous motor neuron degeneration • Microglial inflammatory switch (M2→M1) • Oligodendrocyte metabolic failure	**Microglia:** TREM2, NF-κB, NLRP3, NOX2-TNF-α/IL-1 **Astrocytes:** EAAT2 reduction, SOD1 toxicity **Oligodendrocytes:** MCT1 lactate transport failure	**Tier 2** [216,217,246,247,252,253,257,272,273,275] **Tier 3** [224,227,228,229,230,235,239,244] **Tier 4** [222,225,226,232,234,240,242,245,248,249,250,251,254,255,258,259,260,261,264,266,267,268,269,270,274]

## Data Availability

No new data were created or analyzed in this study. Data sharing is not applicable to this article.

## Abbreviations

The following abbreviations are used in this manuscript:

CNS	central nervous system
BBB	blood–brain barrier
EAAT2	excitatory amino acid transporter 2
GLT1	glutamate transporter 1
GABA	gamma-aminobutyric acid
PDGF	platelet-derived growth factor
LIF	leukaemia inhibiting factor
NT	neurotrophin
CNTF	ciliary neurotrophic factor
AD	Alzheimer’s disease
ECs	endothelial cells
PD	Parkinson’s disease
MS	Multiple Sclerosis
HD	Huntington’s disease
mHTT	mutant huntingtin
ALS	Amyotrophic Lateral Sclerosis
MSNs	medium spiny neurons
IGF1	insulin-like growth factor 1
TNF	tumor necrosis factor
IL	interleukin
TGFβ	transforming growth factor beta
BDNF	brain-derived neurotrophic factor
Aβ	amyloid beta
NFTs	neurofibrillary tangles
TREM2	triggering receptor expressed on myeloid cells 2
ROS	reactive oxygen species
RAGE	receptor for advanced glycation end products
HMGB1	high mobility group box 1
NF-κB	nuclear factor kappa-light-chain-enhancer of activated B cells
C1q	complement component 1q
Nuk	neural kinase
MMPs	matrix metalloproteinases
VEGF-A	vascular endothelial growth factor A
ACE2	angiotensin-converting enzyme 2
TLR4	toll-like receptor 4
OPC	oligodendrocyte progenitor cell
MHC	major histocompatibility complex
CCL2	monocytic chemotactic protein 1
CXCL10	interferon gamma-induced protein 10
VCAM-1	vascular cell adhesion protein 1
EAE	experimental autoimmune encephalomyelitis
AQP4	aquaporin-4
GM-CSF	granulocyte-macrophage colony-stimulating factor
ICAM-1	intercellular adhesion molecule 1
CSF1R	colony-stimulating factor 1 receptor
IFN-γ	interferon gamma
FGF	fibroblast growth factors
GLAST	glutamate transporter
SOD1	zinc-copper superoxide dismutase 1
MCT1	monocarboxylate transporter 1
